# The impact of paclitaxel and carboplatin chemotherapy on the autonomous nervous system of patients with ovarian cancer

**DOI:** 10.1186/s12883-016-0710-4

**Published:** 2016-10-01

**Authors:** Emmanouil V. Dermitzakis, Vasilios K. Kimiskidis, George Lazaridis, Zoi Alexopoulou, Eleni Timotheadou, Alexandros Papanikolaou, Ourania Romanidou, George Georgiadis, Konstantine T. Kalogeras, Iakovos Tsiptsios, Basil Tarlatzis, George Fountzilas

**Affiliations:** 1 Laboratory of Clinical Neurophysiology, Department of Neurology, “Papageorgiou” Hospital, Thessaloniki, 564 03 Greece; 2 Laboratory of Clinical Neurophysiology, Aristotle University of Thessaloniki, Faculty of Medicine, Thessaloniki, Greece; 3 Department of Medical Oncology, School of Health Sciences, Faculty of Medicine, “Papageorgiou” Hospital, Aristotle University of Thessaloniki, Thessaloniki, Greece; 4 Department of Biostatistics, Health Data Specialists Ltd, Athens, Greece; 5 First Department of Obstetrics and Gynecology, School of Health Sciences, Faculty of Medicine, “Papageorgiou” Hospital, Aristotle University of Thessaloniki, Thessaloniki, Greece; 6 Neurological Department, “Hippocration” General Hospital, Thessaloniki, Greece; 7 Laboratory of Molecular Oncology, Hellenic Foundation for Cancer Research/Aristotle University of Thessaloniki, Thessaloniki, Greece; 8 Translational Research Section, Hellenic Cooperative Oncology Group, Data Office, Athens, Greece

**Keywords:** 30/15 ratio, Autonomous nervous system, Orthostatic hypotension, Paclitaxel, Sympathetic skin response

## Abstract

**Background:**

Paclitaxel-based regimens are frequently associated with the development of peripheral neuropathy. The autonomous nervous system (ANS) effects, however, of this chemotherapeutic agent remain unexplored.

**Methods:**

We investigated a group of 31 female patients with ovarian cancer receiving treatment with paclitaxel and carboplatin, as well as a group of 16 healthy age- and gender-matched healthy volunteers. All study participants completed a questionnaire and were assessed neurophysiologically at three time points (baseline, 3–4 months and 6–8 months following the onset of chemotherapy). The evaluation of the ANS included assessment of the adrenergic cardiovascular function (orthostatic hypotension-OH), parasympathetic heart innervation (30/15 ratio) and sympathetic skin response (SSR).

**Results:**

At the 3–4 months ANS assessment, 19.2 % of the patients had systolic OH and the same percentage had diastolic OH, but at the 6–8 months evaluation no patient had systolic OH and only 13.8 % had diastolic OH. The values of the 30/15 ratio were significantly reduced at both time points, whereas the SSR was not affected.

**Conclusions:**

Combined paclitaxel and carboplatin chemotherapy is associated with significant effects on the parasympathetic heart innervation and occasionally with effects on the adrenergic cardiovascular reaction. The SSR remained unaffected. Physicians should be alert to the possibility of these treatment-emergent side effects, so as to monitor ANS parameters and introduce treatment modifications accordingly. Our findings however, should be validated in larger cohorts.

**Electronic supplementary material:**

The online version of this article (doi:10.1186/s12883-016-0710-4) contains supplementary material, which is available to authorized users.

## Background

Paclitaxel is commonly used as first-line chemotherapy for advanced ovarian cancer and as adjuvant treatment, in combination with cisplatin or carboplatin, for residual disease. Taxanes (paclitaxel and docetaxel) are associated with numerous side effects, particularly including neurotoxic phenomena [1] . For instance, severe peripheral neuropathy is known to occur in patients receiving cumulative doses of around 1000 mg/m^2^ paclitaxel and 400 mg/m^2^ docetaxel [2]. In recent years, research interest focused on maximizing the therapeutic efficacy of paclitaxel, while minimizing the associated side effects. Despite these efforts, primarily sensory and occasionally sensorimotor [3, 4] peripheral neuropathy occurs in 59 to 78 % of treated patients [5–7].

Generalized peripheral neuropathies are frequently accompanied by autonomous nervous system (ANS) dysfunction [8]. However, it is currently unknown whether paclitaxel or its combination with carboplatin affects the ANS and whether paclitaxel-induced neuropathy comprises autonomic phenomena, as well.

The present study was designed to address this issue and investigate the effects of the combination of paclitaxel and carboplatin chemotherapy on the ANS. From a clinical point of view, paclitaxel is occasionally associated with hypotension, bradycardia and hypertension. Accordingly, we investigated the impact of paclitaxel on the sympathetic and parasympathetic innervation of the heart and performed additional tests to assess sudomotor function (i.e. Sympathetic Skin Response–SSR) and somatic peripheral nerve fibers (i.e. sensory and motor Nerve Conduction Velocities–NCVs). It should be noted that the above tests are easily performed in the context of a routine clinical neurophysiological examination and were employed to increase the utility and relevance of our findings to every-day clinical practice.

## Methods

### Patients’ selection

Thirty-one female patients with ovarian cancer receiving treatment with paclitaxel and carboplatin entered the study. The therapeutic regimen involved six sessions of combined chemotherapy administered every 3 weeks in the following manner: paclitaxel was given at a dose of 175 mg/m^2^ as a 3-h i.v. infusion (with monitoring of blood pressure and pulses every hour) and carboplatin was administered at a dose estimated from the formula Dose (mg) = target AUC (mg/ml.min) x [GFR (ml/min) + 25]. A control group comprising 16 age- and gender-matched healthy volunteers was also included.

Exclusion criteria included age <18 years, history of peripheral neuropathy, intake of drugs affecting the ANS, alcohol abuse, diabetes mellitus and previous exposure to chemotherapy.

The clinical protocol was approved by the Institutional Review Board of ‘Papageorgiou’ Hospital and the Bioethics Committee of the Aristotle University of Thessaloniki. All study participants provided written informed consent for participating in the study, which was in accordance with the ethical standards laid in the 1964 Declaration of Helsinki.

### Procedures

For the assessment of ANS function, participants completed a questionnaire and underwent neurophysiological examination of the ANS and motor and sensory NCVs, by the same neurologist. All tests took place in the morning, in a quiet environment. Study participants were asked to fast, refrain from smoking and exercise for the previous 12 h and sleep comfortably 8 h the night before. Patients had to have no symptoms of sepsis and were examined at least 7 days after their last cycle of chemotherapy. Patients underwent ANS testing and completed an ANS and neuropathy questionnaire (Additional file 1) at three time points: at baseline (*n* = 31), 3–4 months (*n* = 26) and 6–8 months (*n* = 29) after the first chemotherapy cycle. Healthy volunteers (*n* = 16) were examined once.

### Questionnaire

All study participants completed the same questionnaire at all visits. The total number of positive responses for the ANS before and after chemotherapy administration was used as a subjective measure of the effect of paclitaxel in combination with carboplatin on the patients’ quality of life.

### Neurophysiological examination

The assessment of the ANS and somatic peripheral nerve fibers was performed as previously reported [9]. Briefly, ANS testing included the assessment of the parasympathetic heart innervation by measuring alterations in cardiac rhythm in response to changes in posture from the supine to the erect position, known as the 30/15 ratio. The adrenergic cardiovascular function was assessed by measuring alterations in blood pressure (BP) in response to changes in posture from the supine to the standing position. Orthostatic hypotension (OH) was defined as a systolic BP (SBP) drop of ≥20 mmHg and/or a diastolic BP (DPB) drop of ≥10 mmHg within 3 min of active standing [10]. Sympathetic sudomotor function was evaluated using the SSR test [11]. NCVs were also examined in order to diagnose or monitor the evolution of a sensory and/or motor neuropathy, the severity of which was determined on the basis of the Common Terminology Criteria for Adverse Events (NIH-NCI, 2003, version 3.0).

### Statistical analysis

Categorical parameters were presented as counts with corresponding percentages, while for continuous parameters various measures were used (median plus range and/or mean plus range or standard deviation (SD)). Variable distributions were tested for normality by the use of Kolmogorov-Smirnov test. Abnormal values among the evaluation time points (baseline, 3–4 and 6–8 months) were tested for marginal homogeneity using the McNemar’s test [12]. Measurements between baseline and post-treatment assessments were compared by the use of Wilcoxon signed ranks tests [13]. The Mann-Whitney test [14] was used for the comparison of the median 30/15 ratio with neuropathy grade (0–I vs. II–III). Progression-free survival (PFS) was measured from the time of initiation of first-line treatment until verified disease progression, death or last contact (whichever occur first), while, overall survival (OS) was measured from the time of initiation of first-line treatment until death from any cause or date of last contact. All tests were two-sided with level of significance set at 5 %. The SPSS software (version 15.0, IBM Corporation, Armonk, NY, USA) was used for statistical analysis.

## Results and discussion

The mean age in the patient group was 52 years (range 38–74) and in the healthy control group 54 years (range 28–77). Five patients decided not to participate for personal reasons in the 3–4 month assessment and two in the 6–8 month assessment without, however, interrupting or modifying the overall treatment schedule. Table 1 summarizes the number of chemotherapy cycles, median dose intensities and cumulative doses for the two administered drugs while Table 2 includes survival data (progression-free and overall survival) for the patient group.

**Table 1:** Number of chemotherapy cycles, median dose intensities and median cumulative doses. Combined paclitaxel and carboplatin chemotherapy was administered to the 31 ovarian cancer patients

Total number of cycles administered	195
Median number of cycles (range)	6 (4–8)
Median cumulative dose (mg) (range)	
Paclitaxel	1737 (1108–2664)
Carboplatin	3915 (1550–6220)
Median dose intensity (mg/m^2^/week) (range)	
Paclitaxel	164.3 (109.2–247.8)
Carboplatin	396.0 (141.1–558.9)

**Table 2:** Survival data for the patient group (*n* = 31)

Progression-free survival (PFS)	
Progressions [*n* (%)]	14 (45.2)
3-year PFS (%)	61.3
Range (months)	6.2–47.8
Overall survival (OS)	
Deaths [n (%)]	9 (29.0)
3-year OS (%)	79.8
Range (months)	14.9–52.9
Median follow-up (months)	39.7

### ANS assessments

Autonomous nervous system assessment results did not differ significantly between the patient and healthy control groups at baseline.

At the 3–4 months ANS assessment, the number of patients with orthostatic hypotension (OH) increased compared to baseline. After 3 min of active standing, reduction in systolic blood pressure (SBP) of ≥20 mmHg wasn’t observed in any patient at baseline but in five patients (19.2 %) at 3–4 months. A reduction in diastolic blood pressure (DBP) of ≥10 mmHg was observed in two patients (7.7 %) at baseline but on five patients (19.2 %) at the 3–4 months evaluation. These differences, however, failed to reach statistical significance possibly as a result of a type II error due to small sample size. The values of the 30/15 ratio were significantly reduced at the 3–4 months assessment compared to baseline (Wilcoxon signed ranks test, *P* = 0.016) whereas the SSR was not affected (Table 3).

**Table 3:** Three to four months evaluation of 30/15 ratio and sympathetic Skin Response (SSR)

	Baseline (*n* = 31)		3–4 months evaluation (*n* = 26)		Wilcoxon signed ranks test
	Mean (SD)	Median (Range)	Mean (SD)	Median (Range)	*P*-value
30/15 ratio	1.13 (0.09)	1.10 (1.00–1.43)	1.10 (0.20)	1.03 (0.89–1.94)	**0.016**
SSR					
Upper limbs	1.46 (0.34)	1.38 (1.10–3.01)	1.61 (0.45)	1.53 (1.10–3.01)	0.17
Lower limbs	2.22 (0.42)	2.20 (1.60–3.13)	2.27 (0.47)	2.25 (1.60–3.13)	0.74

*SSR* sympathetic skin response

Significant *p*-values are shown in bold

At the 6–8 month evaluation, 26 patients (89.7 %) among the 29 patients, who were examined, had completed chemotherapy. The results of the orthostatic hypotension testing were clearly improved compared to the 3–4 month assessment. Thus, no patient had a reduction of ≥20 mmHg after 3 min of active standing at the 6–8 months evaluation, similarly to baseline. Four patients (13.8 %) had a reduction in DBP of ≥10 mmHg after 3 min of active standing compared to three patients (9.7 %) at baseline. The values of the 30/15 ratio were significantly reduced compared to baseline (*P* = 0.002) whereas the SSR wasn’t affected (Table 4).

**Table 4:** Six to eight months evaluation of 30/15 ratio and sympathetic Skin Response (SSR)

	Baseline (*n* = 31)		6–8 months evaluation (*n* = 29)		Wilcoxon signed ranks test
	Mean (SD)	Median (Range)	Mean (SD)	Median (Range)	*P*-value
30/15 ratio	1.13 (0.09)	1.10 (1.00–1.43)	1.07 (0.09)	1.06 (0.94–1.30)	**0.002**
SSR					
Upper limbs	1.46 (0.34)	1.38 (1.10–3.01)	1.50 (0.41)	1.46 (1.00–3.01)	0.58
Lower limbs	2.22 (0.42)	2.20 (1.60–3.13)	2.13 (0.41)	2.08 (1.59–3.13)	0.74

*SSR* sympathetic skin response

Significant *p*-values are shown in bold

On an individual patient level, the incidence of abnormal 30/15 ratio varied longitudinally as follows: three patients had abnormal values at both baseline and the 3–4 months time points whereas one patient had an abnormal baseline value which subsequently normalized. Twelve patients had normal values at baseline, which became abnormal at 3–4 months (McNemar’s test, *P* = 0.003), whereas ten patients had consistently normal values. One patient had abnormal value at both baseline and the 6–8 months time point and two patients had abnormal baseline values, which subsequently normalized. Twelve patients had normal values at baseline, which became abnormal at 6–8 months (McNemar’s test, *P* = 0.013), whereas 14 patients had consistently normal values.

At the 6–8 months evaluation, four patients were still on treatment or <1 month after treatment completion and 25 patients had completed treatment more than a month earlier. These two groups were not significantly different in any of the examined ANS parameters. In the latter group of 25 patients, there were no significant differences in ANS parameters between the 3–4 month and the 6–8 month evaluation.

### Assessment of the 30/15 ratio according to the grade of sensory neuropathy

With regard to the severity of sensory neuropathy, patients were dichotomized into two groups: those with no sensory neuropathy or asymptomatic neuropathy only (grade 0–I) and those with higher grade sensory neuropathy (grade II–III). No differences in the median 30/15 ratio were found between the two groups of patients at either post-treatment evaluation (Table 5).

**Table 5:** Incidence of sensory neuropathy and association with the median 30/15 ratio

Sensory neuropathy	*n* (%)	30/15 ratio	Mann-Whitney test (grade 0–I vs. II–III)
		Median (range)	*P*-value
3–4 months evaluation (*n* = 26)			
Grade 0	8 (30.8)	1.10 (0.97–1.31)	0.36
Grade I	2 (7.7)	1.01 (1.00–1.03)	
Grade II	15 (57.7)	1.03 (0.89–1.94)	
Grade III	1 (3.8)	-	
6–8 months evaluation (*n* = 28)			
Grade 0	6 (21.4)	1.12 (1.00–1.30)	0.054
Grade I	3 (10.7)	1.10 (1.00–1.22)	
Grade II	15 (53.6)	1.03 (0.94–1.23)	
Grade III	4 (14.3)	1.00 (1.00–1.14)	

### Questionnaire results

Regarding the incidence of positive responses in the ANS dysfunction questionnaire, there were no significant differences in the distribution of positive answers between healthy volunteers and patients at the baseline evaluation. However, the mean (2.35) and median (2) number of positive answers was higher at the 3–4 months (Wilcoxon signed ranks test, *P* = 0.002) but not the 6–8 months (mean = 1.66 and median = 2, *P* = 0.33) evaluations compared to baseline (mean = 1.29 and median = 1).

The present study was designed to investigate the ANS effects of paclitaxel in conjunction with carboplatin in patients with ovarian cancer. It is concluded that the intravenous administration of paclitaxel at a dose of 175 mg/m^2^ in combination with carboplatin affects the sympathetic heart innervation, resulting in primarily systolic orthostatic hypotension during the treatment period. In addition, this chemotherapeutic regimen affects parasympathetic heart innervation both during treatment, as well as during the period ensuing after the last chemotherapy session. Finally, this drug combination has minimal effects on the function of the ANS beyond the cardiovascular system.

In our cohort, no patient had systolic orthostatic hypotension at baseline but five out of 26 patients developed this side effect at the 3–4 month assessment. Interestingly, at the 6–8 month evaluation, when the vast majority of patients had completed treatment more than a month earlier, no subject had evidence of systolic orthostatic hypotension. These observations suggest that the effect of combined paclitaxel and carboplatin chemotherapy on the sympathetic heart innervation is short lived and confined during the treatment period only. It should be noted that in a study on the effects of oxalplatin on sympathetic heart innervation in patients with colorectal cancer, the effects were not transient [9]. This difference may possibly be attributed to the mean age of patients participating in the present study being lower.

The ANS effects of combined paclitaxel and carboplatin chemotherapy were even more evident on the parasympathetic heart innervation, as the 30/15 ratio was significantly affected at all time points following the onset of treatment (i.e both at the 3–4 and 6–8 months assessments). However, this protracted decrease of the 30/15 ratio was not associated with the severity of a chemotherapy-induced sensory neuropathy.

The existing literature on the cardiotoxicity of paclitaxel suggests that transient, asymptomatic bradycardia may occur in up to 29 % of treated patients [15]. Cardiac dysfunction may also occur due to combination of paclitaxel with other chemotherapeutics, such as the monoclonal antibody trastuzumab [16] or epirubicin [17, 18]. It is well known that the administration of paclitaxel in conjunction with doxorubicin in patients with breast cancer is cardiotoxic [19–22]. However, in our study none of the patients received such combinations of chemotherapeutic regimens.

It could be argued that the cardiotoxicity observed in the present study may be due, at least in part, to carboplatin, the co-administered chemotherapeutic agent. Several publications have reported potential subtle cardiotoxic effects of carboplatin when administered in combination with paclitaxel [23–25].

The fact that SSR remained unaffected, despite the development of a primarily sensory and to a lesser extent motor peripheral neuropathy in a sizeable proportion of our patients, is not surprising. It is well known that when fast-conducting, large-diameter myelinated fibers are affected (for instance in cases of carpal tunnel syndrome), the SSR remains intact and is not regarded diagnostically useful in this particular setting [26].

The findings from the ANS evaluations corresponded temporally to increases in positive responses in the questionnaire completed by the patients at 3–4 and 6–8 months following the onset of chemotherapy. At baseline, the mean number of positive responses was 1.13, increasing to a maximum of 2.35 positive responses at the 3–4 months assessment and dropping to a mean of 1.66 at the 6–8 months time point, when the majority of patients had completed the treatment regimen. It is conceivable, however, that the increase in positive responses may simply reflect the general malaise induced by chemotherapy, rather than being the clinical expression of treatment side effects specifically induced on the ANS.

## Conclusions

The present study highlights the fact that combined paclitaxel and carboplatin chemotherapy for ovarian cancer is associated with significant effects on the sympathetic and parasympathetic heart innervation, whereas the SSR remains relatively untouched. Physicians caring for patients with ovarian cancer should be alert to the possibility of these treatment-emergent side effects, so as to monitor ANS parameters (BP, cardiac rhythm and their alterations upon standing) and introduce treatment modifications accordingly. Our findings however, should be validated in larger cohorts.

## Additional file

Additional file 1: Questionnaire, Patient and healthy volunteers’ Autonomous Nervous System and neuropathy questionnaire. (DOC 31 kb)

## Abbreviations

ANS: Autonomous nervous system
BP: Blood pressure
DBP: Diastolic blood pressure
NCVs: Nerve conduction velocities
OH: Orthostatic hypotension
OS: Overall survival
PFS: Progression-free survival
SBP: Systolic blood pressure
SD: Standard deviation
SSR: Sympathetic skin response
