# Supplementary material for: The impact of paclitaxel and carboplatin chemotherapy on the autonomous nervous system of patients with ovarian cancer
Source: BMC Neurol. 2016 Oct 1;16:190. doi: 10.1186/s12883-016-0710-4 (PMC5045633; doi:10.1186/s12883-016-0710-4)
Supplement: Additional file 1: — Questionnaire, Patient and healthy volunteers’ Autonomous Nervous System and neuropathy questionnaire. (DOC 31 kb) [file 12883_2016_710_MOESM1_ESM.doc]

**Patient and healthy volunteers’ Autonomous Nervous System and neuropathy questionnaire.**

- Do you feel dizzy, particularly weak or ready to pass out when standing up from lying position?

**□ YES □ NO**

- Do you ever feel butterflies in your chest?
- **□ YES □ NO**
- Do you feel dizzy, particularly weak or ready to pass out after a heavy meal?
- **□ YES □ NO**
- Do you have abdominal floating, heartburn or excessive hiccup?
- **□ YES □ NO**
- Do you feel sick in the heat or that you cannot perspire?
- **□ YES □ NO**
- Do you have constipation or fecal incontinence?
- **□ YES □ NO**
- Do you have nocturnal diarrheas?
- **□ YES □ NO**
- Do you have difficulty urinating or incontinence?
- **□ YES □ NO**
- Does light bother you or do you often have blurred vision?
- **□ YES □ NO**
- Do you feel that your hands and particularly your feet are permanently numb or do you feel as if they are burning after you begun chemotherapy?
- **□ YES □ NO**
- Do you feel weakness in the upper or lower limbs after you begun chemotherapy?
- **□ YES □ NO**
